# Benchmarking the Solubility Enhancement and Storage Stability of Amorphous Drug–Polyelectrolyte Nanoplex against Co-Amorphous Formulation of the Same Drug

**DOI:** 10.3390/pharmaceutics14050979

**Published:** 2022-05-02

**Authors:** Li Ming Lim, Jin-Won Park, Kunn Hadinoto

**Affiliations:** 1 School of Chemical and Biomedical Engineering, Nanyang Technological University, Singapore 637459, Singapore; particletechnology.ntu@gmail.com; 2 Department of Chemical and Biomolecular Engineering, Seoul National University of Science and Technology, Seoul 01811, Korea; jwpark@seoultech.ac.kr

**Keywords:** poorly soluble drug, solubility enhancement, amorphous, curcumin, ciprofloxacin

## Abstract

Amorphization, typically in the form of amorphous solid dispersion (ASD), represents a well-established solubility enhancement strategy for poorly soluble drugs. Recently, two amorphous drug formulations, i.e., the amorphous drug–polyelectrolyte nanoparticle complex (nanoplex) and co-amorphous system, have emerged as promising alternatives to circumvent the issues faced by ASD (i.e., large dosage requirement, high hygroscopicity). In the present work, the nanoplex was benchmarked against the co-amorphous system in terms of the preparation efficiency, drug payload, thermal stability, dissolution rate, supersaturation generation, and accelerated storage stability. Weakly acidic curcumin (CUR) and weakly basic ciprofloxacin (CIP) were used as the model poorly soluble drugs. The CUR and CIP nanoplexes were prepared using chitosan and sodium dextran sulfate as the polyelectrolytes, respectively. The co-amorphous CUR and CIP were prepared using tannic acid and tryptophan as the co-formers, respectively. The benchmarking results showed that the amorphous drug nanoplex performed as well as, if not better than, the co-amorphous system depending on the drug in question and the aspects being compared. The present work successfully established the nanoplex as an equally viable amorphous drug formulation as the more widely studied co-amorphous system to potentially serve as an alternative to ASD.

## 1. Introduction

Approximately 40% of marketed drugs and 70% of newly discovered drugs possess poor aqueous solubility [1], resulting in their low systemic bioavailability upon administration and consequently low therapeutic efficacy. Amorphization represents a well-established solubility enhancement strategy of poorly soluble drugs by virtue of the metastable form of amorphous drugs that results in a lower energy barrier for dissolution compared to crystalline drugs [2]. In addition to faster dissolution, amorphous drugs are capable of generating a highly supersaturated drug concentration, resulting in a drug’s kinetic solubility that is multifold higher than its thermodynamic solubility [3].

A majority of amorphous drugs approved clinically (e.g., Sporanox^®^, Norvir^®^) are marketed in the form of amorphous solid dispersion (ASD), where the amorphous drugs are molecularly dispersed in inert polymers having a high glass transition temperature (e.g., hydroxypropylmethylcellulose (HPMC), polyvinylpyrrolidone (PVP)) [4]. The drug–polymer interactions suppress devitrification by virtue of reduced drug molecular mobility, resulting in enhanced stability of the amorphous drugs during handling, storage, and dissolution [5].

Nevertheless, stable ASD formulations often require a high mass fraction of the polymer due to limited drug–polymer miscibility [6]. The high polymer’s mass fraction in ASD leads to a large dosage requirement to achieve the desired therapeutic dose. Moreover, the high polymer’s mass fraction also causes ASD to be highly hygroscopic due to the inherent hygroscopicity of the polymers used, resulting in their challenging handling and processing [7,8]. For this reason, alternative amorphous drug formulations that can circumvent the issues of large dosage requirement and handling/processing of ASD have been actively investigated. Examples of such formulations are the co-amorphous (CAM) system, mesoporous silica-based ASD, amorphous nanoparticles, and hybrid systems combining different amorphization strategies [9,10,11].

Among them, the CAM system stands out as one of the most extensively studied amorphous drug formulations besides ASD, which could be attributed to its relatively similar preparation method to ASD (e.g., spray drying, melt-quench, ball milling) [12]. The CAM system circumvents the limited drug–polymer miscibility issue by using low molecular weight compounds (e.g., amino acid, organic acid, or secondary drug) as the stabilizing co-formers, in place of polymers. The low molecular weight of the co-former results in stronger intermolecular interactions with the amorphous drugs (e.g., hydrogen bond, ionic interactions), thereby a lower co-former’s mass fraction is needed in the CAM system compared to in ASD [13].

Importantly, stabilization of amorphous drugs in CAM systems is also evident in the absence of intermolecular interactions between the drug and the co-former. Molecular mixing between the drug and the co-former often suffices for amorphous stabilization in CAM systems [14]. Numerous CAM systems have been successfully developed from a wide range of poorly soluble drugs and co-formers [9]. Bioavailability enhancement afforded by CAM systems has been demonstrated in vivo in several studies [12].

Besides the CAM system, amorphous drug–polyelectrolyte nanoparticle complex (or nanoplex in short) represents another promising alternative to ASD, particularly for weakly acidic and basic poorly soluble drugs [15]. The amorphous nanoplex is attractive owed to its (i) high drug payload and (ii) simple preparation method with a minimal energy requirement involving only the mixing of the drug and polyelectrolyte (PE) solutions under ambient conditions. The resultant nanoplex suspension is then spray-dried or freeze-dried if the nanoplex is intended for oral solid dosage form. A wide range of drugs and PE have been successfully formulated into an amorphous nanoplex [16,17,18,19,20,21,22] and in vivo bioavailability enhancements have been demonstrated in several studies [23,24,25,26]. Importantly, the amorphous nanoplex fares well in benchmarking studies against ASD [27,28].

The amorphous drug–PE nanoplex is formed by electrostatic binding between ionized drug molecules and oppositely charged PE to produce soluble drug–PE complexes. The drug–PE complexes aggregate among themselves due to inter-drug hydrophobic interactions between the bound drug molecules. The drug–PE complexes precipitate out of the solution to form the drug–PE nanoplex upon reaching a critical aggregate mass, whose value is dictated by the drug’s hydrophobicity. The drug–PE electrostatic binding restricts the drug’s molecular mobility to rearrange into ordered crystalline structures upon precipitation, resulting in the formation of amorphous drugs. The drug–PE electrostatic binding is also responsible for the stability of the drug nanoplex during handling and storage. Polymers used in ASD (e.g., HPMC, PVP) are often added to the nanoplex formulation to improve its storage stability and supersaturation generation [29].

Despite the nanoplex’s attractive characteristics, the popularity of CAM systems far exceeds that of the nanoplex, as deduced from their respective numbers of publications in the literature (keywords: “co-amorphous drugs” yielded >175 hits compared to <50 hits for “amorphous drug nanoplex” from Web of Science in February 2022). While both CAM and nanoplex systems have been widely demonstrated to be effective, a direct comparison between these two emerging amorphous systems on their solubility enhancement and storage stability has never been carried out. Previous studies in which different amorphous drug formulations were examined typically either compared different amorphization methods for the same amorphous system (usually ASD) [30,31] or benchmarked new amorphous drug formulations against ASD or pure amorphous drugs [27,32]. Recognizing the CAM system as the frontrunner to replace ASD, we believe a benchmarking study of the nanoplex against the CAM system can shine a light on the feasibility of the nanoplex as an effective amorphous drug delivery system.

**Figure 1:**
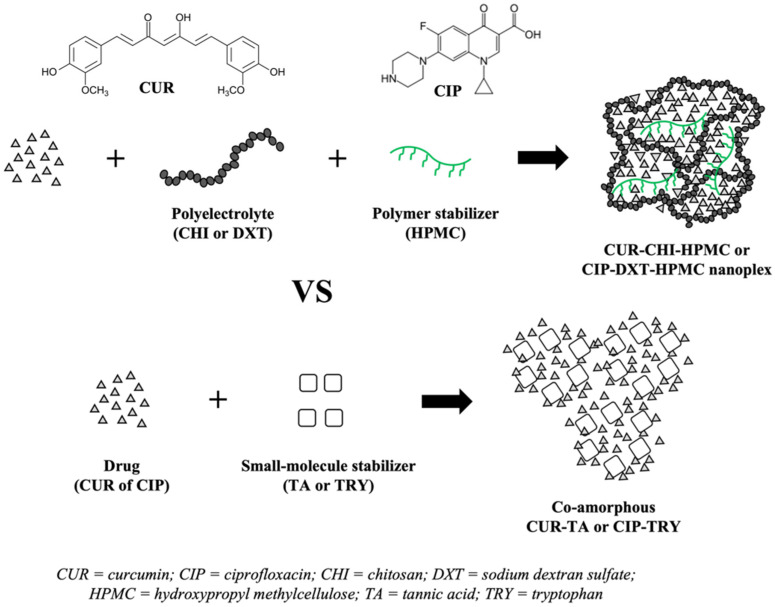
Comparison of amorphous drug nanoplex versus co-amorphous system using two model poorly soluble drugs, i.e., CUR and CIP.

The present work examined the physical characteristics and performances of the CAM and nanoplex systems intended for formulation as oral solid dosage forms. The CAM and nanoplex systems were examined in terms of their (1) drug payload, (2) morphology, (3) preparation efficiency (i.e., drug utilization rate, overall yield), (4) thermal stability, (5) dissolution rate, (6) in vitro solubility enhancement, and lastly, (7) accelerated storage stability. The study as depicted in Figure 1 was carried out using two model poorly soluble drugs, i.e., weakly acidic curcumin (CUR) and weakly basic ciprofloxacin (CIP). The CUR and CIP nanoplexes were prepared using chitosan (CHI) and sodium dextran sulfate (DXT) as the oppositely charged PEs, respectively. Polymer HPMC was incorporated into the nanoplexes to enhance their storage stability [17,18].

**Figure A1:**
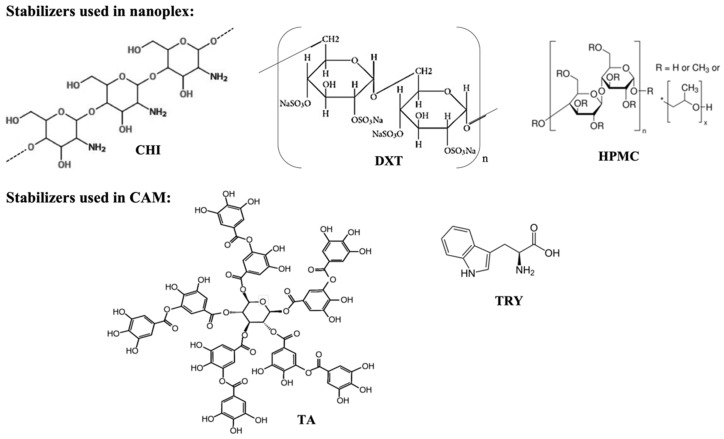
Chemical structures of the constituents of the nanoplex and CAM systems, i.e., CHI, DXT, HPMC, TA, and TRY.

CAM systems of CUR and CIP were prepared using an organic acid, i.e., tannic acid (TA), and amino acid, i.e., tryptophan (TRY), as the co-formers, respectively, following the works of Ke et al. [33] and Zhu et al. [34], respectively. The stabilization of amorphous drugs in the co-amorphous CUR–TA and CIP–TRY was afforded by hydrogen bond interactions between the acids and the drugs. The chemical structures of TA and TRY presented in Figure A1 of Appendix A show the abundant existence of hydrogen bond donor groups (particularly for TA, owed to its abundant catechol and pyrogallol groups) to interact with hydrogen bond acceptor groups of CUR (e.g., phenol, carbonyl) and CIP (e.g., carboxyl, pyridone).

## 2. Materials and Methods

### 2.1. Materials

CUR from turmeric rhizome (≥95% curcuminoid content) and CIP (≥98%) was purchased from Alfa Aesar (Singapore, Singapore) and TCI Chemicals (Tokyo, Japan), respectively. Sodium dextran sulfate (DXT) MW = 5 kDa was purchased from Wako Pure Chemical (Tokyo, Japan). CHI (190–310 kDa, 75–85% deacetylation), tannic acid (TA), tryptophan (TRY), hydroxypropyl methylcellulose (HPMC) (26 kDa), potassium hydroxide (KOH), potassium dihydrogen phosphate (KH_2_PO_4_), sodium chloride (NaCl), ethanol, methanol, acetonitrile, glacial acetic acid, and 4-(2-hydroxyethyl)-1-piperazineethanesulfonic acid (HEPES) were purchased from Sigma-Aldrich (Singapore, Singapore).

### 2.2. Methods

#### 2.2.1. Preparation of CUR-CHI-HMPC and CIP-DXT-HPMC Nanoplexes

The CUR–CHI–HPMC nanoplex was prepared following the methods described in Lim, et al. [18]. Briefly, 5 mg/mL CUR and 6 mg/mL of HPMC were dissolved in 0.01 M KOH (pH 12). CUR having pK_a_ of 8.4, 9.9, and 10.5 [35] was fully deprotonated at pH 12 to form anionic CUR molecules. Separately, 6.4 mg/mL CHI was dissolved in 1.2% (*v/v*) aqueous acetic acid solution (pH 2.7). CHI having a pK_a_ of 6.5 [35] was protonated at pH 2.7 to form cationic CHI molecules. The (CUR + HPMC) solution was added immediately after its preparation to the CHI solution at an equal volume (5 mL each) under gentle stirring. The resultant CUR–CHI–HPMC nanoplex was ultrasonicated for 20 s at 20 kHz (VC 505, Sonics, Oklahoma City, OK, USA). The nanoplex suspension was washed by two cycles of centrifugation (14,000× *g*, 10 min) to remove excess CUR, CHI, and HPMC, followed by re-dispersion in deionized water.

The CIP–DXT–HPMC nanoplex was prepared following the methods described in Dong, et al. [17]. Briefly, 5 mg/mL CIP was dissolved in 0.4% (*v/v*) aqueous acetic acid solution (pH 3.0). CIP having a pKa of 6.1 and 8.6 [36] was fully protonated at pH 3.0 to form cationic CIP molecules. Separately, 3 mg/mL DXT and 5 mg/mL HPMC were dissolved in deionized water. DXT having a pKa < 2 [29] was deprotonated at neutral pH to form anionic DXT molecules. The CIP solution was added to the (DXT + HPMC) solution at an equal volume (5 mL each) under gentle stirring. The resultant CIP–DXT–HPMC nanoplex suspension was washed following the same procedures described above to remove excess CIP, DXT, and HPMC. The CUR–CHI–HPMC and CIP–DXT–HPMN nanoplex suspensions were lyophilized for 24 h (Alpha 1–2 LDPlus, Martin Christ, Osterode am Harz, Germany) at −52 °C and 0.05 mbar to produce nanoplex powders for characterizations.

#### 2.2.2. Preparation of Co-Amorphous CUR-TA and CIP-TRY

The co-amorphous CUR–TA was prepared by pH-shift nanoprecipitation using TA as the stabilizer following the method described in Ke et al. [33]. Briefly, 1 mg/mL CUR was dissolved in 0.01 M KOH (pH 12) and 1 mg/mL TA was dissolved in 50 mM HEPES solution (pH 5.2) after which both solutions were mixed at equal volume (5 mL) under vortexing. The resultant CUR–TA particle suspension (pH 6.6) was washed to remove excess CUR and TA, and subsequently lyophilized by the same procedures described above.

The co-amorphous CIP–TRY was prepared by adopting the lyophilization method of Zhu et al. [34], who prepared co-amorphous TRY and ofloxacin, which is chemically similar to CIP as they are both fluoroquinolone antibiotics. Briefly, CIP was dissolved at 0.5 mg/mL in 0.5 mg/mL aqueous TRY solution. The CIP–TRY solution was then syringe filtered using a sterile syringe filter with a 0.22 μm pore size PVDF membrane (Merck-Millipore, Burlington, MA, USA) to ensure the removal of any non-soluble CIP. Afterwards, the filtered CIP–TRY solution was lyophilized for 48 h to produce CIP–TRY powders. For reference runs, physical mixtures of CIP and TRY and CUR and TA were prepared at the same drug: co-former ratios exhibited by the CAM systems.

#### 2.2.3. Preparation Efficiency

The preparation efficiency of the nanoplex and CAM systems was characterized by the drug utilization rate and overall yield defined in Equations (1) and (2), respectively. For the two nanoplexes and co-amorphous CUR–TA, the mass of drug that formed the nanoplex or CAM in Equation (1) was determined from a minimum of six replicates by calculating the difference between the initial mass of drug added and the mass of drug recovered in the supernatant after the first centrifugation cycle.

(1) $$\mathrm{Drug}\;\mathrm{utilization}\;\mathrm{rate}\;\left(\%\;w/w\right)=\frac{\mathrm{Mass}\;\mathrm{of}\;\mathrm{drug}\;\mathrm{that}\;\mathrm{formed}\;\mathrm{nanoplex}\;\mathrm{or}\;\mathrm{CAM}}{\mathrm{Initial}\;\mathrm{mass}\;\mathrm{of}\;\mathrm{drug}\;\mathrm{added}}\times 100$$

The mass of CUR in the supernatant was determined by high-performance liquid chromatography (HPLC) (Agilent 1100, Agilent Technologies, Santa Clara, CA, USA) at a detection wavelength of 423 nm using 80% (*v/v*) aqueous ethanol solution as the mobile phase at 1.0 mL/min in a ZORBAX Eclipse Plus C18 column (250 × 4.6 mm, 5 µm particle size), resulting in a CUR retention time of ≈2.5 min. The mass of CIP in the supernatant was determined by HPLC in the same ZORBAX column at a detection wavelength of 324 nm using 0.1 M KH_2_PO_4_: acetonitrile: methanol solution (60:25:15% *v/v*) as the mobile phase at 1 mL/min, resulting in CIP retention time of ≈5 min.

For the co-amorphous CIP–TRY, the mass of the drug that formed CAM in Equation (1) was determined from six replicates by determining, using HPLC, the mass of CIP recovered in the lyophilized CIP–TRY powders after their dissolution in 1.2% (*v/v*) acetic acid solution. For the yield calculation, the mass of the nanoplex or CAM produced in Equation (2) was determined from six replicates from the dry mass of the lyophilized nanoplex and CAM powders.

(2) $$\mathrm{Yield}\;\left(\%\right)=\frac{\mathrm{Mass}\;\mathrm{of}\;\mathrm{nanoplex}\;\mathrm{or}\;\mathrm{CAM}\;\mathrm{produced}}{\mathrm{Initial}\;\mathrm{masses}\;\mathrm{of}\;\mathrm{drug}\;\mathrm{and}\;\mathrm{excipients}\;\mathrm{added}}\times 100$$

#### 2.2.4. Physical Characterizations

The drug payload defined in Equation (3) was determined from six replicates by dissolving the lyophilized nanoplex or CAM powders in 80% (*v/v*) ethanol solution for the CUR powders or in 1.2% (*v/v*) acetic acid solution for the CIP powders. Subsequently, the mass of CUR and CIP in the solution was determined by HPLC as described previously.

(3) $$\mathrm{Payload}\;\left(\%\;w/w\right)=\frac{\mathrm{Mass}\;\mathrm{of}\;\mathrm{drug}\;\mathrm{in}\;\mathrm{nanoplex}\;\mathrm{or}\;\mathrm{CAM}}{\mathrm{Total}\;\mathrm{mass}\;\mathrm{of}\;\mathrm{nanoplex}\;\mathrm{or}\;\mathrm{CAM}}\times 100$$

For both the nanoplexes and the co-amorphous CUR–TA, their size, polydispersity index (PDI), and zeta potential prior to lyophilization were determined in triplicates using a Brookhaven 90 Plus Nanoparticle Size Analyzer (Brookhaven Instruments Corporation, Holtsville, NY, USA). Their morphology was examined by a field emission scanning electron microscope (FESEM) (JSM 6700F, JEOL, Peabody, MA, USA) using lyophilized powders as the representative sample. For the co-amorphous CIP-TRY, the size was characterized by image analysis using ImageJ software (NIH, Bethesda, MD, USA) with a minimum of 300 particle counts, and the morphology was examined by a scanning electron microscope (SEM) (6390LA, JEOL, USA). Species interactions in the nanoplex and CAM systems were examined by Fourier transform infrared spectroscopy (FTIR) using a Spectrum One FT-IR spectrometer (PerkinElmer, Hopkinton, MA, USA) performed between 500 and 4000 cm^−1^ at 1 cm^−1^ spectral resolution.

The amorphous forms of the nanoplex and CAM systems were examined by powder X-ray diffraction (PXRD) using a D8 Advance X-ray Diffractometer (Bruker, Berlin, Germany) performed between 10° and 70° (2θ) with a step size of 0.02° and scanning rate of 1.2°/min. PXRD analysis was also performed for physical mixtures of (1) the drugs and the PEs (i.e., CHI, DXT) and (2) the drugs and the co-formers (i.e., TA, TRY) prepared at the same drug:PE (or co-former) ratios exhibited by the nanoplex (or CAM). The thermal stability was examined by thermal gravimetric analysis (TGA) (Pyris Diamond TGA, PerkinElmer, USA) and differential scanning calorimetry (DSC) (DSC 822E, Mettler Toledo, Columbus, OH, USA). The TGA analysis was performed at a heating rate of 10 °C/min between 25 °C and 400 °C, whereas the DSC analysis was performed at a heating rate of 2 °C/min between 25 °C and 300 °C.

#### 2.2.5. Dissolution Profile under Sink Condition

The dissolution rates of the drugs from the nanoplex and CAM powders were characterized in simulated intestinal juice (SIJ) under sink conditions herein defined as ¼ of the thermodynamic solubility of the drugs (C_Sat_). The SIJ was prepared by adjusting 0.9% (*w/v*) aqueous KH_2_PO_4_ solution to pH 6.8 by the addition of 0.2 M KOH. C_Sat_ of CUR and CIP in the SIJ was determined in triplicates prior to the dissolution testing. Briefly, excess CUR or CIP was incubated in excess in the SIJ over 24 h at 37 °C in a shaking incubator. Afterwards, the solution was centrifuged to remove undissolved CUR or CIP after which the drug concentration in the supernatant was determined by HPLC.

In addition, C_Sat_ of CUR in the SIJ containing 0.01 to 0.1 mg/mL of CHI and TA and C_Sat_ of CIP in the SIJ containing 0.1 to 1 mg/mL of DXT and TRY were determined to assess the influence of the PEs and co-formers, if any, on the C_Sat_ of CUR and CIP. Using the above protocols, C_Sat_ of CUR and CIP in the SIJ was determined to be equal to 4.9 ± 1.4 and 120 ± 6 μg/mL, respectively. The effects of the PEs and co-formers on C_Sat_ were found to be insignificant as the variations in C_Sat_ due to the presence of the PEs and co-formers fell within the experimental uncertainties.

For the dissolution testing, the lyophilized nanoplex or CAM powders were added at ¼ C_Sat_ to 100 mL SIJ maintained at 37 °C in a shaking incubator. Next, 1 mL of aliquot was withdrawn at specific time points over 1 h. Fresh SIJ of the same volume was added back to the dissolution vessel as replenishment. Afterwards, the aliquot was centrifuged at 14,000× *g* for 3 min and then syringe filtered (0.22 μm pore size PVDF membrane (Merck-Millipore, USA)). The drug concentration in the filtered supernatant was determined by HPLC as previously described.

As CUR is known to undergo hydrolytic degradation at intestinal pH [37], the chemical stability of CUR in the SIJ (pH 6.8) was characterized in triplicates. Briefly, the native CUR was fully dissolved in 100 mL of 95:5 (*v/v*) SIJ:ethanol at the same concentration used in the dissolution testing (i.e., ¼ of 4.9 μg/mL). The resultant CUR solution was incubated in a shaking incubator for 24 h at 37 °C h during which 1 mL of aliquot was withdrawn after 3, 6, 9, 12, and 24 h. The CUR concentration in the aliquot was quantified by HPLC from which the half-life of CUR in the SIJ was determined.

#### 2.2.6. Supersaturation Generation

The drug solubility enhancements afforded by the nanoplex and CAM systems were characterized by the supersaturation generation reported here as the ratio of the supersaturated drug concentration (C) to C_Sat_. Briefly, the lyophilized nanoplex and CAM powders were added in excess at 8 × C_Sat_ to 40 mL SIJ and placed in a shaking incubator maintained at 37 °C. Next, 400 µL of aliquot was withdrawn and syringe filtered (0.22 μm pore size PVDF membrane (Merck-Millipore, USA)) at specific time points over 3 h and 6 h for CUR and CIP, respectively. The aliquot was immediately diluted tenfold with fresh SIJ to prevent drug precipitation from the supersaturated solution. The drug concentrations in the aliquot were determined by HPLC as previously described. The dissolution rate and supersaturation generation of the native crystalline CUR and CIP were characterized for comparison.

#### 2.2.7. Accelerated Storage Stability

The lyophilized nanoplex and CAM powders were stored in an open container inside a desiccator for three months under an accelerated storage condition (i.e., 40 °C and 75% relative humidity). The three-month accelerated storage simulated approximately twelve-month storage under normal conditions (i.e., 25 °C and 60% relative humidity) [38]. The 75% relative humidity was generated inside the desiccator by placing an open container of saturated NaCl solution at 40 °C. The amorphous forms of the nanoplex and CAM powders were examined by PXRD after one month and three months of accelerated storage. The storage stability was also examined using DSC as it could detect the formation of crystals from the drug payload and dissolution profile of the stored samples.

## 3. Results and Discussion

### 3.1. Benchmarking Nanoplex against CAM System

#### 3.1.1. Preparation Efficiency

For CUR, the CUR–CHI–HPMC nanoplex exhibited a significantly higher CUR utilization rate at 84 ± 2% (*w/w*) than the co-amorphous CUR-TA at 39 ± 4% (*w/w*) (Table 1) indicating that a large proportion of CUR in the feed (>80%) was successfully transformed to the nanoplex. The higher CUR utilization rate in the CUR–CHI–HPMC nanoplex could be attributed to the strong electrostatic interactions between CUR and CHI that led to the neutralization of CUR charges, resulting in CUR precipitation as nanoplex. Whereas the CUR–TA formation relied solely on the shift in the environment pH from alkaline to neutral upon mixing of the CUR and TA solutions, which caused CUR to be insoluble.

For the same reason, the CIP utilization rate in the CIP–DXT–HPMC nanoplex was comparably high at 81 ± 5% (*w/w*), owed to strong electrostatic interactions between CIP and DXT. The co-amorphous CIP–TRY, on the other hand, exhibited a nearly 100% CIP utilization rate, owed to its distinct preparation method where a saturated CIP solution in TRY was used as the starting material. Thereby, nearly all CIP in the feed (after filtration) could be lyophilized and recovered as the final product. This co-amorphization method was feasible for CIP–TRY because CIP, being a weakly basic drug, was soluble in the presence of a weakly acidic co-former in TRY.

Despite the >80% drug utilization rates, the overall preparation yields of the CUR–CHI–HPMC and CIP–DXT–HPMC nanoplexes were lower than 50% (Table 1). The relatively low yields were inevitable because the nanoplex was typically prepared at excess PE to drug charge ratios to ensure sufficient PE charges available for complexation with the charged drug molecules. The presence of excess HPMC that was not incorporated into the nanoplex further contributed to the lowering of the yield. For the CAM systems, the yield of the co-amorphous CUR–TA was expectedly lower (34 ± 3% *w/w*) than that of the CUR–CHI–HPMC nanoplex due to its lower CUR utilization rate. Not surprisingly, the yield of the co-amorphous CIP–TRY was significantly higher at 91 ± 4% (*w/w*) due to the nature of its preparation method as previously discussed.

On this note, while it was true that the preparation yield was highly dependent on the initial masses of drugs, PEs, and co-formers used, it was worth pointing out that the initial masses used could not be arbitrarily increased or decreased from the present values without affecting the product characteristics (e.g., drug payload, size). In fact, in the case of the nanoplex, using different initial drug and PE concentrations might not result in the nanoplex formation at all. Therefore, the preparation yield reported herein essentially represented the optimal preparation yield for both the nanoplex and CAM systems.

#### 3.1.2. Drug Payload and Morphology

The CUR payload of the CUR–CHI–HPMC nanoplex was designed to be comparable to the CUR payload of the co-amorphous CUR-TA at 55 ± 1% vs. 49 ± 4% *w/w*, respectively (Table 1). Comparable CUR payloads between the two amorphous systems were desired to enable their direct comparison as the drug payload was known to influence the solubility enhancement and storage stability of amorphous systems [39]. The same approach was pursued in the CIP–DXT–HPMC nanoplex and co-amorphous CIP–TRY, resulting in their comparable CIP payloads at 59 ± 4% (*w/w*) and 51 ± 2% (*w/w*), respectively. The comparable drug payloads were achieved by manipulation of the design variables for the nanoplex and CAM system’s preparation (e.g., drug concentration, pH).

With regards to their morphology, the CUR–CHI–HPMC nanoplex, CIP–DXT–HPMC nanoplex, and co-amorphous CUR–TA possessed nanoscale sizes of roughly 300–400 nm as measured by DLS (Table 1). For comparison, the sizes of the native CUR and native CIP were between around 3 and 8 µm based on their microscope images. On this note, the size of the co-amorphous CUR–TA prepared in the present work was considerably larger than the size of the CUR–TA particles prepared in Ke et al. [33] (<70 nm) due to two reasons. First, Ke et al. [33] prepared the CUR–TA particles by flash nanocomplexation in a confined impinging jet mixer, resulting in a much higher supersaturation level (owed to the faster and more uniform mixing) than the supersaturation level generated by the bulk mixing employed in the present work. The higher supersaturation level in turn led to increased CUR nucleation events and consequently suppressed particle growth, resulting in smaller particles produced.

Even when bulk mixing of CUR and TA solutions was employed in [33], they used a much smaller volume at 50 µL compared to the 5 mL used in the present work. The smaller volume used resulted in a superior mixing, hence a higher supersaturation level. Second, in addition to TA, polyvinyl alcohol (PVA) was added as a steric stabilizer in [33] to minimize the agglomeration of the CUR–TA nanoparticles after their formation. In the present work, an additional colloidal stabilizer was not used as the CUR–TA nanoparticles were intended for oral solid dosage form, thereby they were not going to be stored as an aqueous suspension. In the absence of an additional colloidal stabilizer, it was not unexpected that larger CUR–TA nanoparticles were produced in the present work.

Despite the absence of an additional colloidal stabilizer, the co-amorphous CUR–TA was found to exhibit good colloidal stability as evidenced by its high zeta potential of approximately −32 mV (Table 1). The negative zeta potential of CUR–TA was due to TA having a negative charge at neutral pH [40]. Similarly, zeta potentials of the CUR–CHI–HPMC and CIP–DXT–HPMC nanoplexes were also high in the range of (+/−) 32 to 37 mV with PDI < 0.4 indicating good monodispersity. The CUR–CHI–HPMC and CIP–DXT–HPMC nanoplexes exhibited oppositely charged zeta potentials due to the presence of cationic CHI and anionic DXT on their surfaces, respectively.

**Figure 2:**
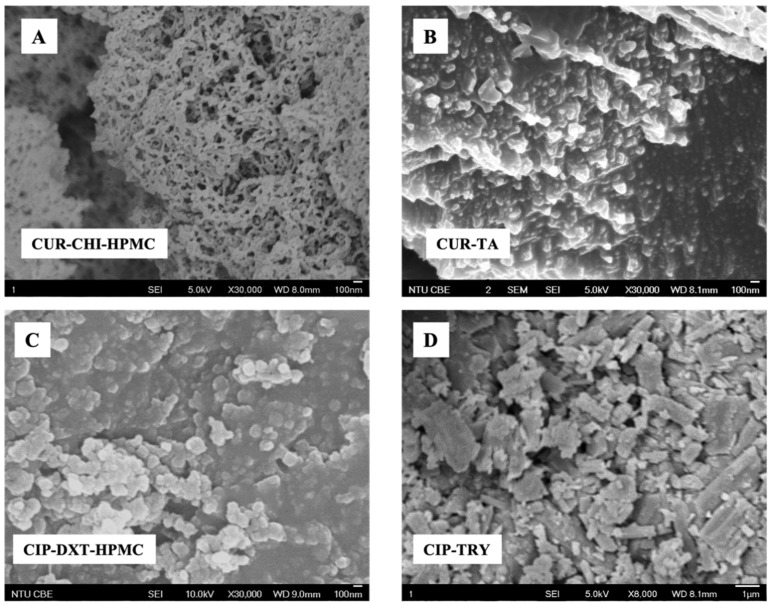
FESEM images of (**A**) CUR–CHI–HPMC nanoplex; (**B**) co-amorphous CUR–TA; (**C**) CIP–DXT–HPMC nanoplex; (**D**) co-amorphous CIP–TRY.

The nanoscale sizes of the CUR–CHI–HPMC and CIP–DXT–HPMC nanoplexes and co-amorphous CUR–TA were confirmed by FESEM analysis, which showed the appearance of roughly spherical nanoparticles (100–200 nm in size) for the CIP–DXT–HPMC nanoplex (Figure 2C) and co-amorphous CUR-TA (Figure 2B). The CUR–CHI–HPMC nanoplex, on the other hand, appeared smaller (<100 nm) with a worm-like shape (Figure 2A). In contrast, the co-amorphous CIP–TRY was significantly larger with a size in the micrometer of 1790 ± 430 nm (Table 1) exhibiting a roughly rectangular shape (Figure 2D). The micrometer size of the co-amorphous CIP–TRY was not unexpected as the CIP–TRY was formed by slow sublimation of its frozen aqueous solution in the freeze dryer.

#### 3.1.3. Species Interaction by FTIR

##### CUR Amorphous Systems

**Figure 3:**
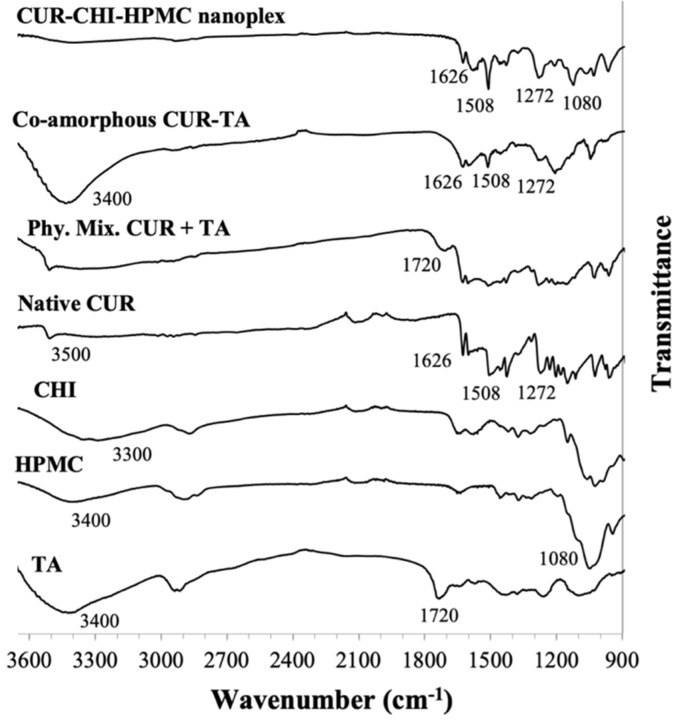
FTIR spectra of CUR-CHI-HPMC nanoplex and co-amorphous CUR-TA.

Detailed FTIR analysis of species interactions in the CUR–CHI–HPMC nanoplex was presented earlier in Lim et al. [18] and summarized briefly here (Figure 3). The presence of CUR in the nanoplex was characterized by the appearance of bands at 1626, 1508, and 1272 cm^−1^ attributed to the stretching vibrations of the C = C−C_ring_, C = O, and enol C-O bonds of the native CUR, respectively. The CUR–CHI electrostatic interactions in the nanoplex were characterized by the disappearance of the phenolic OH band of the native CUR at 3500 cm^−1^ due to its electrostatic interaction with CHI’s charged amine group represented by stretching NH_2_ band at 3300 cm^−1^. The inclusion of HPMC in the nanoplex was evident from the appearance of the band unique to HPMC at 1080 cm^−1^ attributed to its glycosidic C-O-C bond vibration.

Even though co-amorphous CUR–TA has been prepared previously [33], to the best of our knowledge, species interactions in the co-amorphous CUR-TA have not been examined before. The presence of CUR in the co-amorphous CUR-TA was evident from the appearance of the abovementioned native CUR bands at 1626, 1508, and 1272 cm^−1^ (Figure 3). The presence of TA was evident from the strong broad band at 3400 cm^−1^ attributed to the OH stretching of TA’s catechol and pyrogallol groups. The CUR–TA interactions were characterized in the CUR–TA spectrum by the disappearance of the 1720 cm^−1^ band attributed to carbonyl group stretching due to hydrogen bond interactions with CUR. The 1720 cm^−1^ band otherwise appeared in the FTIR spectra of raw TA and physical mixture of CUR and TA. The CUR–TA hydrogen bond interactions were also reflected from the reduced intensity of the carbonyl (1508 cm^−1^) and enol (1272 cm^−1^) bands of CUR.

##### CIP Amorphous Systems

**Figure 4:**
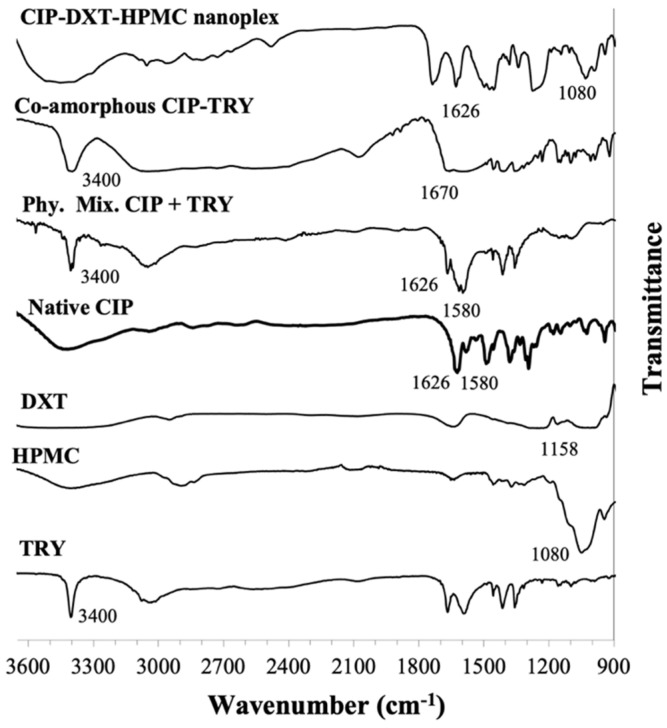
FTIR spectra of CIP-DXT-HPMC nanoplex and co-amorphous CIP-TRY.

Similar to the CUR–CHI–HPMC nanoplex, a detailed FTIR analysis of species interactions in the CIP–DXT–HPMC nanoplex was presented earlier in Dong et al. [17] and is summarized briefly here (Figure 4). The presence of CIP in the nanoplex was characterized by the band at 1626 cm^−1^ attributed to C = O stretching of the pyridone group of the native CIP. The CIP–TRY electrostatic interaction in the nanoplex was characterized by the absence of the 1580 cm^−1^ band attributed to the NH_2_ bending of the piperazine group of the native CIP, as a result of its interaction with the sulfate group of DXT represented by the band at 1158 cm^−1^. The presence of HPMC in the nanoplex was evidenced by the appearance of the 1080 cm^−1^ band unique to HPMC as previously discussed.

For the co-amorphous CIP–TRY, the presence of CIP and TRY was evident, respectively, from the native CIP’s pyridone band at 1680 cm^−1^ and the strong sharp NH stretching band of TRY’s indole ring at 3400 cm^−1^ (Figure 4). The CIP–TRY interactions were characterized by the shift and weakened of the C = O stretching of CIP’s pyridone band from 1626 to 1670 cm^−1^ and the disappearance of the NH_2_ bending of CIP’s piperazine band at 1580 cm^−1^ due to hydrogen bond interactions with TRY. Without the hydrogen bond interactions, the 1626 and 1580 cm^−1^ bands remained visible in the spectrum of the physical mixture of CIP and TRY.

#### 3.1.4. Thermal Stability by TGA and DSC

##### CUR Amorphous Systems

**Figure 5:**
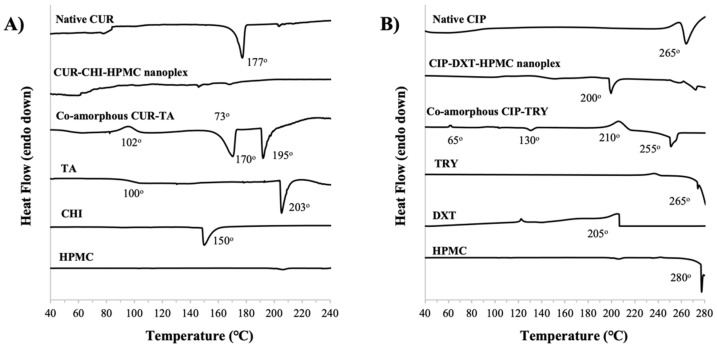
DSC thermographs of (**A**) CUR–CHI–HPMC nanoplex and co-amorphous CUR–TA; (**B**) CIP–DXT–HPMC nanoplex and co-amorphous CIP–TRY.

**Figure A2:**
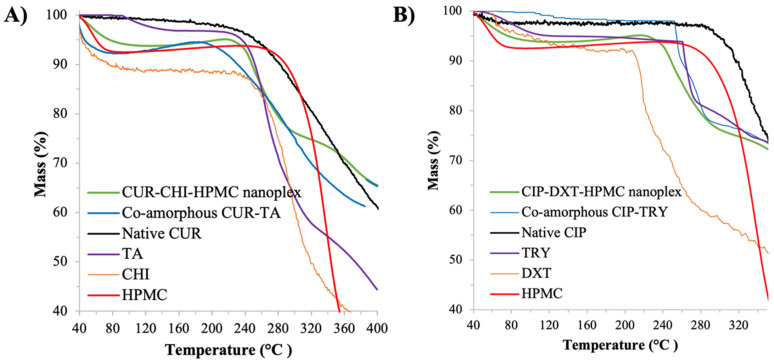
TGA thermographs of (**A**) CUR’s and (**B**) CIP’s nanoplex and CAM systems.

The TGA results showed that CUR and the excipients used (i.e., CHI, HPMC, TA) had largely decomposed at a temperature above 240 °C (Figure A2 of Appendix A). The DSC thermograph below 240 °C showed that the native CUR exhibited an endothermic melting point peak at around 177 °C (Figure 5A) as the native CUR existed as a crystalline solid as shown later by PXRD in Section 3.3.1. The amorphous form of the CUR–CHI–HPMC nanoplex was observed to remain mostly stable upon heating as evidenced by the absence of major exothermic recrystallization and endothermic melting point events in its DSC thermograph. Small endothermic events appearing between 140 °C and 170 °C were attributed to melting events of the CHI constituent of the nanoplex. The DSC thermograph of the raw CHI indeed showed a sharp endothermic peak at 150 °C attributed to the melting points of the crystalline fractions of the native CHI. The DSC thermograph of HPMC, on the other hand, only showed an endothermic event at around 280 °C attributed to HPMC decomposition.

In contrast, the co-amorphous CUR-TA exhibited an exothermic recrystallization event at around 102 °C, followed by sharp endothermic events at 170 °C and 195 °C. The endothermic events were attributed to the crystalline melting point of CUR and melting/decomposition of TA, respectively. The thermal instability of the co-amorphous CUR–TA was therefore triggered by destabilization of its TA constituent as supported by the DSC thermograph of the raw TA that showed a glass transition event at around 100 °C and melting at around 203 °C. In short, the CUR–CHI–HPMC nanoplex exhibited superior thermal stability to the co-amorphous CUR–TA due to the different thermal stability of the excipients used in their formulations.

##### CIP Amorphous Systems

The TGA results showed that CIP had decomposed at around 280 °C, while the excipients used (i.e., TRY, DXT, HPMC) decomposed at significantly lower temperatures, particularly DXT which decomposed at around 205 °C (Figure A2 of Appendix A). The DSC thermographs below 280 °C (Figure 5B) showed that the amorphous CIP–DXT–HPMC nanoplex exhibited good thermal stability upon heating up to 200 °C above which an endothermic event took place, which could be attributed to DXT decomposition. For comparison, the DSC thermograph of the crystalline native CIP showed melting and immediate decomposition events at around 265 °C.

A similar thermal behavior was observed for the co-amorphous CIP–TRY, which remained relatively stable upon heating up to around 210 °C at which a major exothermic recrystallization event began to occur. Upon further heating, an endothermic event took place at around 255 °C due to the melting and decomposition of TRY. In this regard, the DSC thermograph of the raw TRY showed an endothermic melting/decomposition peak starting at around 265 °C, respectively. The DSC thermograph of the co-amorphous CIP-TRY also showed small exothermic and endothermic events at around 65 °C and 130 °C, respectively. In short, the CIP–DXT–HPMC nanoplex and co-amorphous CIP–TRY exhibited similar thermal stability in which both were thermally stable up to approximately 200 °C.

### 3.2. Nanoplex vs. CAM’s Solubility Enhancement

#### 3.2.1. Dissolution Rate

**Figure 6:**
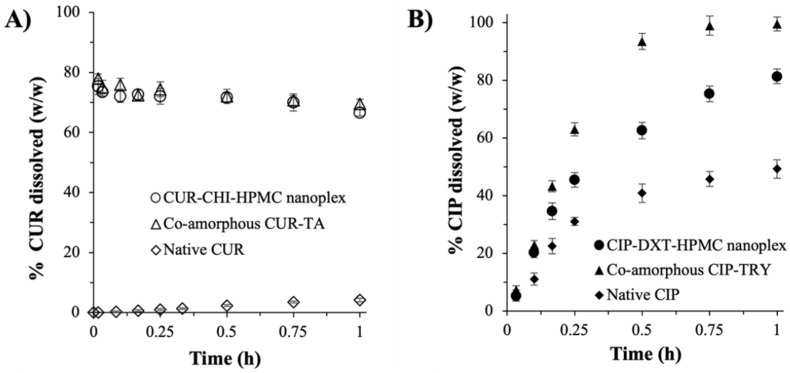
Drug dissolution rates of the nanoplex and CAM systems under sink condition (**A**) CUR and (**B**) CIP.

The CUR–CHI–HPMC nanoplex and co-amorphous CUR-TA exhibited highly comparable dissolution rates under sink conditions (Figure 6A). Burst release profiles in which roughly 75% (*w/w*) of the CUR payload was released under 10 min were observed. The burst release profiles exhibited by the CUR amorphous systems were in contrast to the inhibited CUR release of the crystalline native CUR, where <5% (*w/w*) CUR was released after 1 h due to its extremely low aqueous solubility. The %CUR dissolution from the CUR amorphous systems was observed to slowly decrease after reaching the peak value due to the aforementioned hydrolytic degradation of CUR at the physiological pH of the SIJ (pH 6.8). In this regard, the half-life of CUR in the SIJ under the condition used in the dissolution testing was determined to be between 9 h and 12 h, indicating a slow degradation rate, which was not unexpected as the rate of CUR hydrolytic degradation had been known to intensify at a pH above 7 [37,41].

For the CIP amorphous systems, both the CIP–DXT–HPMC nanoplex and co-amorphous CIP–TRY exhibited comparably fast dissolution rates (Figure 6B). Approximately 45% and 62% (*w/w*) of the CIP payload were released after 15 min for the CIP–DXT–HPMC nanoplex and co-amorphous CIP–TRY, respectively. The %CIP dissolution reached roughly 82% and 99% (*w/w*) after 1 h for the nanoplex and its co-amorphous counterpart, respectively. As expected, the crystalline native CIP exhibited a slower dissolution rate with <50% (*w/w*) dissolution after 1 h. By virtue of the larger surface areas of the nanoplex owed to its nanoscale size, the CIP–DXT–HPMC nanoplex theoretically should lead to a faster dissolution rate than the microscale co-amorphous CIP–TRY. The experimental results, however, did not fit with the theoretical prediction, indicating that the dissolution rates of the CIP amorphous systems were not governed solely by the particle surface areas.

Other factors, such as drug–excipient interactions and physicochemical characteristics of the excipients used, also influenced the drug dissolution. We postulated that the stronger interaction between CIP and DXT in the nanoplex by electrostatic binding than the hydrogen bond interaction between CIP and TRY in the co-amorphous CIP–TRY was the reason behind the latter’s faster dissolution under sink conditions. The use of slowly dissolving large molecular weight DXT and HPMC in the nanoplex versus fast-dissolving small molecule TRY in the co-amorphous CIP–TRY was postulated to be another factor contributing to CIP–TRY’s faster dissolution. Nevertheless, to elucidate the exact mechanisms of how the different drug–excipient interactions and excipients’ physicochemical properties between the nanoplex and CAM systems affected their CIP dissolution profiles, a fundamental study at the molecular level would need to be carried out. This fundamental study, which was beyond the scope of the present work, was similar to how the exact interaction mechanisms between polymer stabilizers (e.g., HPMC, PVP) and drugs in ASD during dissolution are presently being actively investigated by both experimental and molecular dynamic simulation approaches [42,43,44].

#### 3.2.2. Supersaturation Generation

**Figure 7:**
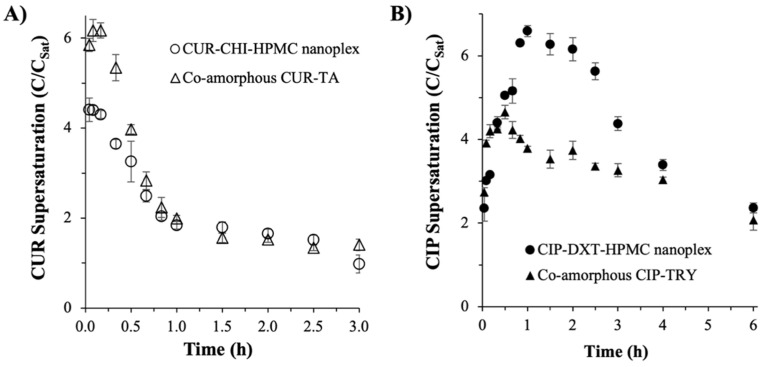
Supersaturation generation of the nanoplex and CAM systems (**A**) CUR and (**B**) CIP.

Both the CUR–CHI–HPMC nanoplex and co-amorphous CUR–TA rapidly dissolved to completion upon their addition in excess at 8 × C_Sat_ to the SIJ. Both of them produced “spring and parachute” supersaturation profiles, where the supersaturation level increased rapidly upon dissolution to reach a peak supersaturation level, followed by a gradual decrease in the supersaturation level due to precipitation of the supersaturated solution, and eventually the supersaturation level went back to the C_Sat_ value. The co-amorphous CUR–TA produced a higher peak supersaturation level at approximately 6.2 × C_Sat_ after 5 min compared to approximately 4.4 × C_Sat_ produced by the CUR–CHI–HPMC nanoplex after the same period (Figure 7A). In both CUR amorphous systems, the peak supersaturation levels gradually decreased with time to settle at roughly 1–1.5 × C_Sat_ after 3 h. Owing to the higher peak supersaturation level, the co-amorphous CUR–TA produced a roughly 13% larger area under the curve (AUC) in the supersaturation versus time profile, signifying its slightly superior solubility enhancement.

Despite the complete dissolution of the drug at the start of the experiment (<2.5 min), which was evidenced by the appearance of a clear solution observed by naked eyes, the peak supersaturation levels were lower than 8 × C_Sat_ added initially, because precipitation of the highly supersaturated CUR solution took place concurrently with the dissolution of the amorphous solids. The net effect of these concurrent dissolution and precipitation events was reflected in the observed supersaturation level.

The higher peak supersaturation level of the co-amorphous CUR–TA could be attributed to the weaker CUR–TA interactions (i.e., hydrogen bonding) compared to the CUR–CHI interactions (i.e., electrostatic binding) in the nanoplex. The weaker CUR–TA interactions were postulated to enable more CUR to dissolve under non-sink conditions in the short time window before precipitation of the highly supersaturated solution took place. Other factors that could contribute to the difference in the supersaturation generation between the two amorphous systems were the physicochemical properties of excipients used (i.e., TA versus CHI, HPMC). As discussed earlier, a fundamental study at the molecular level was needed to elucidate the exact mechanisms of how the physicochemical properties of the excipients used influenced the supersaturation generation.

On this note, the effect of the CUR hydrolytic degradation on the CUR supersaturation profile was deemed minimal because, as we reported earlier, the CUR hydrolytic degradation rate in the SIJ was slow even when the test was carried out under sink conditions in which a very low concentration of CUR was exposed to an abundance of SIJ. Under the sink condition, the CUR concentration in the SIJ (¼ × C_Sat_) was much lower than the CUR concentration present in the supersaturation experiments (8 × C_Sat_). As CUR hydrolytic degradation had been reported to follow first-order kinetics [37], where the degradation rate was independent of the initial CUR concentration, the amount of CUR degraded during the 3 h period of the supersaturation experiments was not expected to greatly influence the supersaturation level.

Similar observations were made in the CIP amorphous systems in which the “spring and parachute” supersaturation profiles were also produced (Figure 7B). Specifically, the CIP–DXT–HPMC nanoplex produced a peak supersaturation level of approximately 6.6 × C_Sat_ after 1 h, followed by a gradual decrease in the supersaturation level to reach 4.4 × C_Sat_ and 2.3 × C_Sat_ after 3 h and 6 h, respectively. The co-amorphous CIP–TRY produced a lower peak supersaturation level of approximately 4.7 × C_Sat_ after 30 min and the supersaturation level gradually decreased to 3.3 × C_Sat_ and 2.1 × C_Sat_ after 3 h and 6 h, respectively.

Both CIP–DXT–HPMC nanoplex and co-amorphous CIP–TRY dissolved to completion upon their addition in excess to SIJ as evidenced by the appearance of a clear solution after 23 ± 7 min and 24 ± 5 min (n = 3) for the CAM and nanoplex systems, respectively. The complete dissolution of the CIP amorphous systems took longer than the CUR amorphous systems due to the former’s slower dissolution rate as shown earlier in Figure 6. The supersaturation level shown in Figure 7B was contributed by both the dissolution of the amorphous solids and the precipitation of the supersaturated CIP solution. The fact that a clear solution was observed after 20–30 min suggested that the precipitation of the highly supersaturated CIP solution was not instantaneous, which could be attributed to the presence of HPMC and TRY as stabilizing agents.

Despite the inclusion of HPMC, the rate of decrease in the supersaturation level of the CIP–DXT–HPMC nanoplex was found to be faster than that of the co-amorphous CIP–TRY. The faster de-supersaturation rate of the nanoplex could be explained by the larger driving force for crystallization of the supersaturated solution at a higher supersaturation level [45]. In other words, the higher the “spring”, the steeper the “parachute” in the supersaturation versus time profile. Overall, the supersaturation versus time profile of the CIP–DXT–HPMC nanoplex produced a roughly 34% larger AUC than that of the co-amorphous CIP–TRY signifying the superior solubility enhancement of the nanoplex. Like the CUR amorphous systems, the superior supersaturation generation of the CIP–DXT–HPMC nanoplex could be attributed to multiple factors, including drug–excipient interactions and physicochemical characteristics of the excipients used.

### 3.3. Nanoplex vs. CAM’s Storage Stability

#### 3.3.1. PXRD and DSC

##### CUR Amorphous Systems

**Figure 8:**
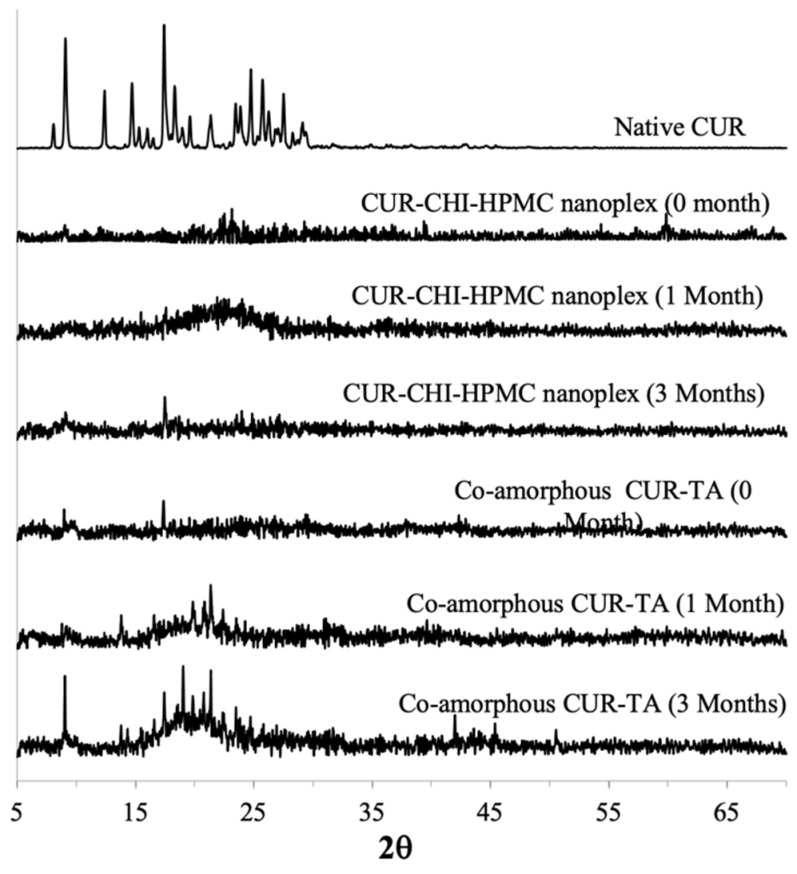
PXRD patterns of CUR–CHI–HPMC nanoplex and co-amorphous CUR–TA after accelerated storage (40 °C and 75% RH) of 0, 1, and 3 months.

**Figure A3:**
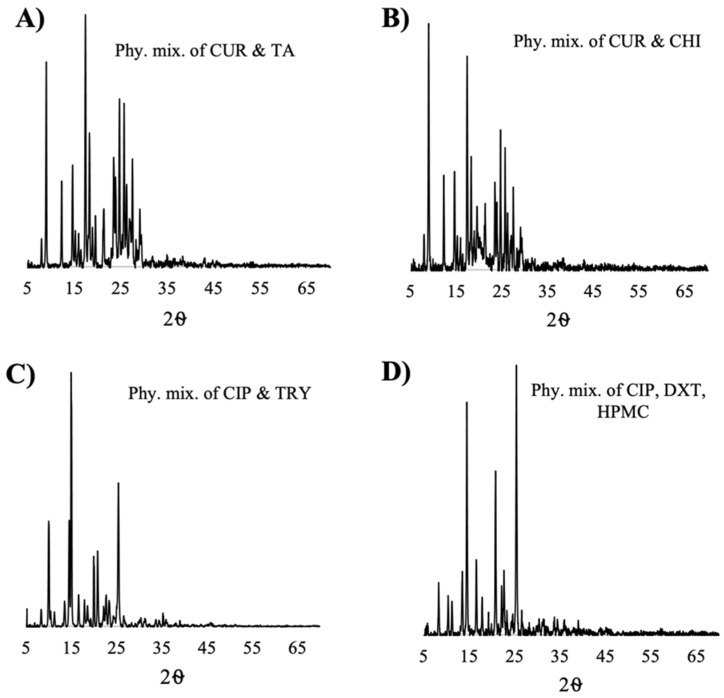
PXRD patterns of the physical mixtures of (**A**) CUR and TA; (**B**) CUR, CHI, and HPMC; (**C**) CIP and TRY; (**D**) CIP, DXT, and HPMC.

The PXRD analysis performed immediately after preparation (0 month) confirmed the amorphous forms of both the CUR–CHI–HPMC nanoplex and co-amorphous CUR–TA. The amorphous forms were evidenced by the absence of high-intensity crystalline peaks in their PXRD patterns, which were observed in the PXRD patterns of the native CUR (Figure 8). High-intensity crystalline peaks were also observed in the physical mixtures of CUR and TA, as well as in the physical mixture of CUR, CHI, and HPMC (Figure A3 of Appendix A). It was worth pointing out that the PXRD pattern of the co-amorphous CUR–TA at 0 month exhibited low-intensity crystalline peaks at roughly 2*θ* = 9 and 17 indicating that the freshly prepared CUR–TA particles were not fully 100% amorphous as it contained small crystallites. The presence of small crystallites in amorphous systems has been known to adversely affect the supersaturation generation (e.g., lower peak supersaturation level, faster de-supersaturation) as well as storage stability [46]. Despite the presence of small crystallites, the supersaturation generation of the co-amorphous CUR–TA was nevertheless superior to that of the CUR–CHI–HPMC nanoplex.

The amorphous form of the CUR–CHI–HPMC nanoplex was found to remain stable after one month of accelerated storage. After three months of accelerated storage, a single medium-intensity peak appeared at 2*θ* = 17 in the PXRD pattern of the nanoplex indicating an amorphous-to-crystalline transformation beginning to take place. Other than that, the amorphous form of the CUR–CHI–HPMC nanoplex was largely maintained after three months of accelerated storage. In contrast, the PXRD pattern of the co-amorphous CUR–TA began to show a few medium-intensity peaks after 1 month of accelerated storage at 2*θ* = 21–24. The peaks intensified after three months of accelerated storage, particularly at 2*θ* = 9, 19, and 22, indicating increased amorphous-to-crystalline transformation while the rest remained largely amorphous. In short, the CUR–CHI–HPMC nanoplex exhibited superior storage stability to the co-amorphous CUR–TA, which was partly contributed by the presence of small crystallites in the co-amorphous CUR–TA.

##### CIP Amorphous Systems

**Figure 9:**
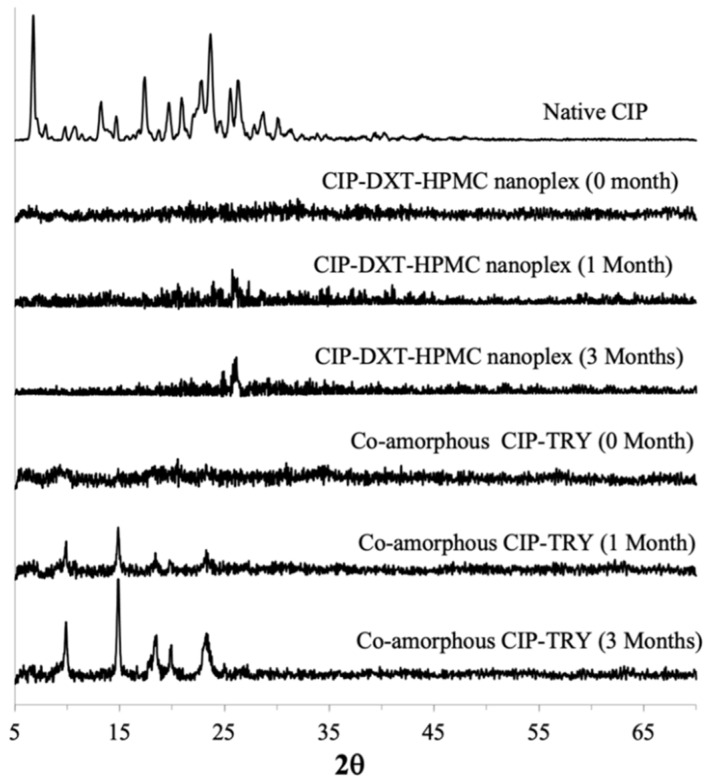
PXRD patterns of CIP–DXT–HPMC nanoplex and co-amorphous CIP–TRY after accelerated storage (40 °C and 75% RH) of 0, 1, and 3 months.

The amorphous forms of the CIP–DXT–HPMC nanoplex and co-amorphous CIP–TRY immediately after their preparation (0 month) were confirmed by the absence of high-intensity peaks in their PXRD patterns, in contrast to the PXRD pattern of the native CIP (Figure 9). High-intensity crystalline peaks were also observed in the physical mixtures of CIP and TRY, as well as in the physical mixture of CIP, DXT, and HPMC (Figure A3 of Appendix A). Similar to the co-amorphous CUR–TA, the PXRD pattern of the co-amorphous CIP–TRY at 0 month exhibited a low-intensity crystalline peak at around 2*θ* = 20 indicating that the freshly prepared CIP–TRY particles were not fully 100% amorphous as it contained small crystallites. We postulated that the presence of the small crystallites in the co-amorphous CIP–TRY was one of the reasons for its inferior supersaturation generation compared to the CIP–DXT–HPMC nanoplex.

**Figure A4:**
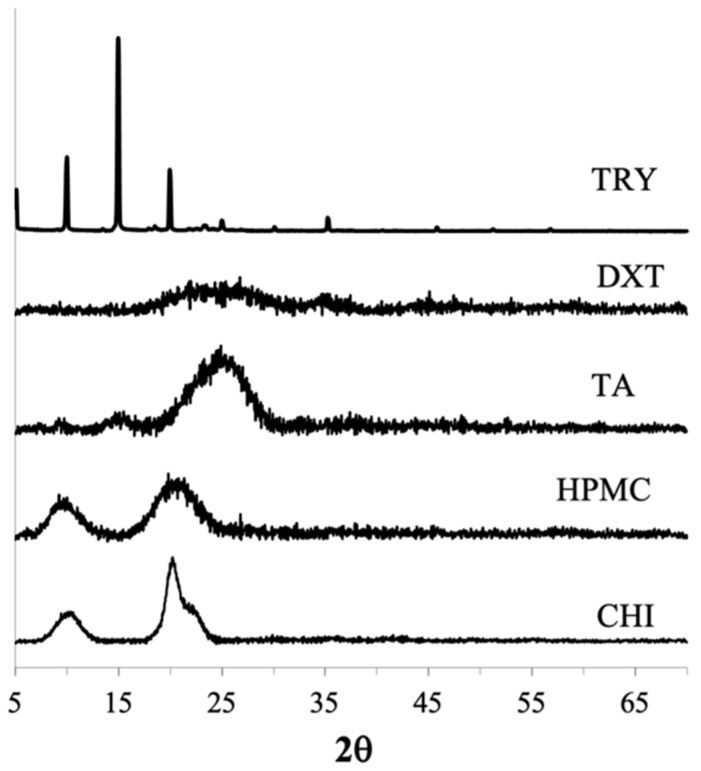
PXRD patterns of TRY, TA, CHI, DXT, HPMC.

The CIP–DXT–HPMC nanoplex remained fully amorphous after one month of accelerated storage. The nanoplex, for the most part, maintained its amorphous form after three months of accelerated storage. Nevertheless, a peak at 2*θ* = 26–27 began to take form indicating an amorphous-to-crystalline transformation was taking place. The co-amorphous CIP–TRY, on the other hand, exhibited an obvious amorphous-to-crystalline transformation after one month of accelerated storage as evidenced by the appearance of medium-intensity peaks at 2*θ* = 10 and 15. The amorphous-to-crystalline transformation intensified after three months with high-intensity peaks appearing at 2*θ* = 10, 15, 18, 20, and 24. The high-intensity peaks at 2*θ* = 10, 15, and 24 were also present in the PXRD pattern of the raw TRY (Figure A4 of Appendix A) indicating both CIP and TRY underwent an amorphous-to-crystalline transformation during the accelerated storage. Hence, similar to the results of the CUR amorphous systems, the CIP nanoplex system exhibited superior storage stability to the CAM system of CIP, which was also partly caused by the presence of small crystallites in the co-amorphous CIP–TRY.

In this regard, the inclusion of a well-established crystallization inhibitor (i.e., HPMC) in the nanoplexes was believed to be the reason for their superior storage stability compared to the CAM systems. The inclusion of HPMC was also believed to play a role in minimizing the formation of small crystallites in the nanoplexes. Our previous studies showed that the binary CUR–CHI and CIP–DXT nanoplexes prepared without HPMC were not stable after three months of accelerated storage [17,18]. Therefore, their storage stability was on par with the co-amorphous CUR–TA and CIP–TRY investigated in the present work. Recently, a number of restudies have begun exploring the benefits of incorporating crystallization-inhibiting polymers, such as HPMC, on the storage stability of ternary CAM systems made up of the drug, co-former, and crystallization-inhibiting polymers [10,47]. How the ternary nanoplex system fared against the ternary CAM system merits an investigation in the future.

**Figure 10:**
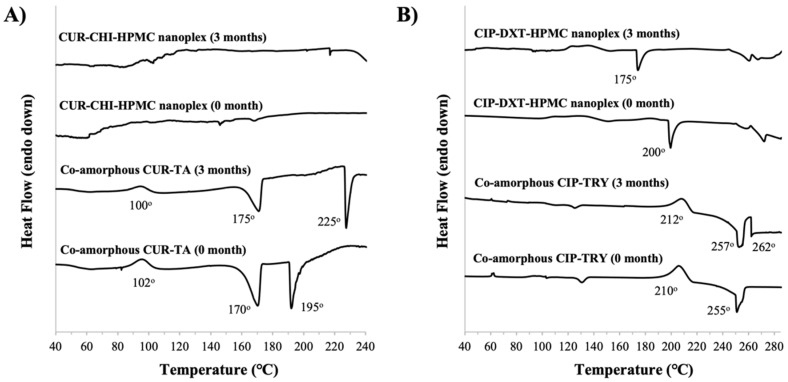
DSC thermographs of the nanoplex and CAM systems of (**A**) CUR and (**B**) CIP before and after three months of accelerated storage.

The storage stability of the CUR and CIP amorphous systems was further examined by DSC analysis of the stored samples. The DSC thermograph of the CUR–CHI–HPMC nanoplex after three months of accelerated storage did not show the appearance of an endothermic melting point peak of CUR around 170–180 °C indicating the stored nanoplex remained largely amorphous in agreement with the PXRD results (Figure 10A). For the co-amorphous CUR–TA, the DSC thermographs of the co-amorphous CUR–TA before and after storage were found to be relatively similar, albeit the shifts in the endothermic peaks to lower temperatures for the stored sample. Thus, there was not a significant change in the solid state of the CUR–TA sample after storage, which was consistent with the PXRD results that only showed the appearance of a few medium-intensity peaks after storage.

The DSC analysis of the stored samples was also carried out for the CIP amorphous systems (Figure 10B). The DSC thermograph of the CIP–DXT–HPMC nanoplex after three months of accelerated storage showed the absence of the endothermic melting point peak of CIP at around 270 °C. Therefore, the CIP–DXT–HPMC remains largely amorphous in agreement with the PXRD results. The DSC thermographs of the co-amorphous CIP–TRY before and after storage were found to be relatively similar, where recrystallization and melting/decomposition events of TRY were observed at around 210–212 °C and 255–257 °C, respectively. Nevertheless, an endothermic event at around 262 °C appeared in the thermograph of the stored sample, which was not evident in the freshly prepared sample’s thermograph. The endothermic event at 262 °C could be attributed to the melting/decomposition of crystalline CIP that was formed during storage as indicated earlier by the PXRD results.

#### 3.3.2. Drug Payload and Dissolution

**Figure A5:**
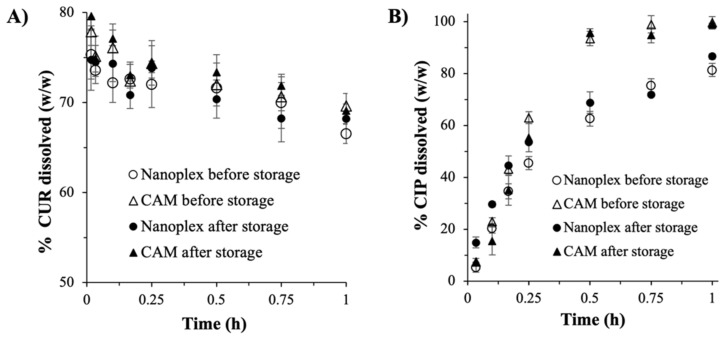
Dissolution profiles before and after storage (**A**) CUR amorphous systems; (**B**) CIP amorphous systems.

The storage stability of the CUR and CIP amorphous systems was assessed further from the drug payloads of the stored samples. After three months of accelerated storage, the CUR payloads of the CUR–CHI–HPMC nanoplex and co-amorphous CUR–TA were not found to change significantly from the CUR payloads before storage (Table 2). The numerical variations in the CUR payloads were within the experimental uncertainties (n = 3). The same findings were reported for the CIP payloads of the CIP–DXT–HPMC nanoplex and co-amorphous CIP–TRY. The small variations in the drug payloads after storage indicated that both amorphous systems remained chemically stable. The chemical stability of the nanoplex and CAM amorphous systems was further demonstrated by the dissolution results of the stored samples, which also showed insignificant variations compared to the freshly prepared samples (n = 3) (Figure A5 of Appendix A).

## 4. Conclusions

The amorphous drug–PE nanoplex was benchmarked against the CAM system of the same drug (i.e., CUR or CIP) in terms of its (i) preparation efficiency, (ii) thermal stability, (iii) dissolution rate under sink conditions, (iv) supersaturation generation, and (v) long-term storage stability. The benchmarking study revealed that the drug nanoplex could perform as well as, if not better than, the CAM system. Depending on the preparation method used, the CAM system could exhibit higher or lower preparation efficiency than the nanoplex. Both CAM and nanoplex systems exhibited good thermal stability, where they remained stable up to 200 °C for the CIP amorphous systems and CUR nanoplex, while up to 100 °C for the co-amorphous CUR. The CAM and nanoplex systems produced comparable fast dissolution rates with good solubility enhancement capabilities characterized by prolonged supersaturation generation at a high level. The differences between the nanoplex and CAM systems were more pronounced when CIP was used as the model drug, particularly in terms of the dissolution rate, supersaturation generation, and storage stability. One aspect in which the nanoplex was clearly superior to the CAM system independent of the drug studied was the storage stability. This was attributed to the inclusion of a crystallization inhibiting polymer in the nanoplex formulation, which was not carried out in the CAM system. In conclusion, the present work successfully established the amorphous drug–PE nanoplex as an equally viable candidate as the more popular CAM system to serve as an alternative to ASD. Further studies using a diverse range of poorly soluble drugs will be carried out to reaffirm the present findings.

## Figures and Tables

**Table 1:** Preparation efficiency and physical characteristics of nanoplex versus CAM systems.

Characteristics	CUR–CHI–HPMC Nanoplex	Co-Amorphous CUR-TA	CIP–DXT–HPMC Nanoplex	Co-Amorphous CIP–TRY
Drug utilization (% *w/w*)	84 ± 2	39 ± 4	81 ± 5	94 ± 1
Yield (% *w/w*)	46 ± 1	34 ± 3	48 ± 3	91 ± 3
Drug payload (% *w/w*)	55 ± 1	49 ± 4	59 ± 4	51 ± 2
Size (nm)	326 ± 31	331 ± 76	298 ± 12	1790 ± 430
PDI	0.22 ± 0.06	0.34 ± 0.06	0.36 ± 0.03	NA
Zeta potential (mV)	32.6 ± 1.1	−36.7 ± 1.4	−32.1 ± 0.8	NA

**Table 2:** Effect of three months of accelerated storage on the drug payloads.

Drug Payload (% *w/w*)	CUR–CHI–HPMC Nanoplex	Co-Amorphous CUR–TA	CIP–DXT–HPMC Nanoplex	Co-Amorphous CIP–TRY
Before storage	55 ± 1	49 ± 4	59 ± 4	51 ± 2
After storage	56 ± 4	53 ± 6	56 ± 5	47 ± 4

## Data Availability

Not applicable.
